# Physical activity, air pollution, and incident long-term conditions: a prospective cohort study

**DOI:** 10.1186/s12916-025-04338-x

**Published:** 2025-08-22

**Authors:** Minshan Huang, Jonathan R. Olsen, Stewart G. Trost, Carlos Celis-Morales, Jill P. Pell, Frederick K. Ho

**Affiliations:** 1 School of Health and Wellbeing, University of Glasgow, Clarice Pears Building, 90 Byres Road, Glasgow, G128TB UK; 2 Institute for Social Science Research, The University of Queensland, Brisbane, Australia; 3 School of Human Movement and Nutrition Sciences, The University of Queensland, Brisbane, Australia; 4 School of Cardiovascular and Metabolic Health, University of Glasgow, Glasgow, UK; 5 Human Performance Lab, Education, Physical Activity and Health Research Unit, University Católica del Maule, Talca, Chile; 6 Centro de Investigación en Medicina de Altura (CEIMA), Universidad Arturo Prat, Iquique, Chile

**Keywords:** Physical activity, Air pollution, Interaction

## Abstract

**Background:**

Physical activity (PA) is consistently associated with lower risk of long-term conditions. Preliminary evidence suggested the associations could be modified by air pollution. This study aims to examine whether air pollution levels modify the associations of PA with all-cause mortality and incident cancer, major adverse cardiovascular events (MACE), type 2 diabetes, and chronic obstructive pulmonary disease (COPD).

**Methods:**

A total of 414,644 UK Biobank participants were included in the analyses. PA was self-reported and objectively measured using accelerometers. PA was self-reported with IPAQ and objectively measured using accelerometers. Annual PM_2.5_, PM_10_, and NO air pollutant concentrations in 2010 were measured using a European land use regression model. Cox proportional hazard models were used to examine the associations of PA and air pollution with health outcomes. Multiplicative and additive interactions were estimated.

**Results:**

During the study period from 2006 to 2022, 31,765 (7.7%) died, 70,299 (17.0%) had incident cancer, 25,130 (8.5%) had type 2 diabetes, 33,284 (8.0%) had MACE, and 18,844 (4.5%) had COPD. Lower PA was associated with higher risk of all health outcomes. Higher concentration of PM_2.5_ was associated with all outcomes except for cancer. The associations of self-reported PA with mortality and cancer were stronger in areas with higher air pollution with significant additive and multiplicative interactions. There was no evidence of moderation for objectively measured PA.

**Conclusions:**

In the UK, air pollution should not be a factor inhibiting the promotion of PA.

**Supplementary Information:**

The online version contains supplementary material available at 10.1186/s12916-025-04338-x.

## Background

Physical activity (PA) is modifiable lifestyle factor associated with all-cause mortality and a wide range of long-term conditions [1–4], and causality has been largely confirmed through randomised controlled trials [5, 6] and Mendelian randomisation studies [7, 8]. However, there has been preliminary evidence suggesting that air pollution may be a modifying factor in the association between PA and health outcomes [9]. Studies have shown heterogenous results with PA being associated with higher respiratory disease risk [10] but lower lung function [11], in areas with higher air pollution. In a South Korean study, increasing PA level over time in areas with higher air pollution was found to be associated with higher cardiovascular disease risk. However, several studies in Denmark have shown consistent associations where PA was associated lower risk of health outcomes irrespective of air pollution [12–14].

It is possible that the conflicting results reflect different levels of exposure to air pollution, but there has been no clear evidence from area with lower base air pollution apart from Denmark. In addition, important limitations exist in the current evidence base. It is known that people living in more deprived areas were disproportionally exposed to air pollutants [15] and were more likely to develop long-term conditions [16], suggesting possible confounding by social deprivation. Moreover, all existing evidence is based on self-reported PA data, which is subject to reporting bias [17], and was found to be less sensitive in predicting health outcomes due to regression dilution bias [18]. There have also been little data on air pollutants other than fine particulate matter (PM_2.5_) [9].

In order to address these limitations, we conducted a prospective cohort study using both self-reported and accelerometer-measured PA to examine whether the association of PA with all-cause mortality and the incidence of four relevant long-term conditions was modified by the concentrations of multiple air pollutants. Based on the Danish studies, we hypothesised that air pollution does not modify the association between PA and health outcomes.

## Methods

### Study design and participants

A prospective cohort study was conducted using UK Biobank data. A total of 502,366 participants were recruited between 2006 and 2010. The ages of the participants ranged from 37 to 73 years old. Participants attended one of 22 assessment centres across England, Scotland, and Wales. At the assessment centre, all participants underwent face-to-face interviews, completed a self-administered touchscreen questionnaire, and underwent a physical examination (including height, weight, and blood pressure) conducted by trained staff. Participants’ ethnicity, smoking status, and alcohol intake were all self-reported. Comorbidities and medical history were obtained from self-report of physician diagnoses and verified by staff during face-to-face interviews. This study was conducted under project 71,392.

### Measurements

#### Outcome variables: death and long-term conditions

The outcomes of the study were all-cause mortality and incident long-term conditions: major adverse cardiovascular disease (MACE), chronic obstructive pulmonary disease (COPD), and type 2 diabetes (T2D). Death certificates were obtained from the National Health Service Information Centre (England and Wales) and the Scottish National Health Service Central Register (Scotland). Hospital admission records were obtained from the Health Episode Statistics (England and Wales) and Scottish Morbidity Records (Scotland). All linkages were undertaken by the UK Biobank and detailed procedures can be found at http://content.digital.nhs.uk/services. Mortality data were available up to November 2022 at the time of analysis and hospital admission data were available up to October 2022 in England, August 2022 in Scotland, and May 2022 in Wales. Therefore, analysis of all-cause mortality and disease-specific outcomes were censored respectively at these dates or date of death if this occurred earlier. International Classification of Diseases Tenth Revision (ICD-10) codes were used to ascertain incidence of long-term conditions (hospitalisations or deaths) in this study. MACE was defined as incident myocardial infarction, heart failure, or stroke (ICD-10 codes: I11.0, I21, I42.0, I42.6–42.7, I42.9, I50, I60–64); COPD using ICD-10 codes J41–44 and J47; T2D using ICD-10 code E11; and cancer using ICD-10 codes C00–C97. Cancer registry was not used for this study because the data was outdated when the analysis was conducted.

#### Exposure variable: physical activity

Self-reported physical activity (PA) data were collected by UK Biobank via a touchscreen questionnaire, using the International Physical Activity Questionnaire (IPAQ) Short Form [19] to provide information on the frequency, intensity and duration of walking, moderate and vigorous activity. Data processing followed the guidelines published by IPAQ [19]. The volume of PA was calculated by applying weights to the time spent on activities of different intensities to produce total physical activity in MET (metabolic equivalent of task)-hour/week.

Accelerometer-measured PA was obtained using Axivity AX3 wrist-worn triaxial accelerometers worn by 103,686 UK Biobank participants between 2013 and 2015. Participants who provided an email address to UK Biobank were invited at random [20]. The dominant wrist of each individual was used over a period of 7 days at 100 Hz, as has been described elsewhere [20]. The 7161 participants with insufficient wear time (< 72 h), missing data, or poor device calibration were excluded. A two-step approach based on random forest and raw acceleration was used to categorise time spent in light, moderate, and vigorous PA based on a method described previously [21, 22]. In this study, the time spent in intensity-specific PA were combined to produce total MET-hour/week as 1.5 * LPA + 4 * MPA + 8 * VPA, where LPA, MPA, and VPA were weekly hour spent in light, moderate, and vigorous intensity PA.

#### Moderator variable: air pollution

Based on the European Study of Cohorts for Air Pollution Effects (ESCAPE) protocol, annual outdoor air pollutant concentrations at home addresses of cohort participants in 2010 were estimated using a European land use regression model, including PM ≤ 10 µm (PM_10_), PM ≤ 2.5 µm (PM_2.5_), PM 2.5–10 µm (PM_coarse_), PM_2.5_ filter absorbance, nitrogen oxides (NO_x_), and nitrogen dioxide (NO_2_) [23, 24]. PM_2.5_ filter absorbance was calculated using the measured filter reflectance using the ISO (International Standardization Organization) 9835 (1993) formula. Absorbance has previously been found to be highly correlated with elemental carbon [25]. Land use regression models are a statistical method that uses multiple linear regression to analyse the association between pollutant concentrations measured at multiple monitoring points and predictors such as traffic, land use, and topography [24]. Separate models had previously been conducted for PM and NO_x_. The PM model included measurements taken between 2008 and 2011 from 20 places in Europe with 20–40 monitoring sites each [26]. These included two areas (39 sites) in the UK: Manchester and London/Oxford. Nitrogen oxides (NO_2_ and NO_x_) were measured, between 2008 and 2011, in 20 European study regions, and NO_x_ was measured in 36 regions, including three areas (119 sites) in the UK: Manchester, London/Oxford, and Bradford [24]. The measurements were validated with acceptable leave-one-out-cross-validation root mean squared error, e.g. < 1.4 μg/m^3^ for PM_2.5_ [23] and < 6.6 μg/m^3^ for NO_2_ [24]. The overall *R*^2^ of the models were very good, e.g. 75%–87% for NO_2_ [24]. We excluded 33,935 UK Biobank residents living in addresses for which pollution exposure could not be modelled.

#### Covariates

Prior knowledge and existing literature [27–31] were used to select potential confounders to include as covariates: age, sex, ethnicity, Townsend area deprivation index, smoking status, alcohol consumption, and diet quality. Age, sex, ethnicity, smoking status, alcohol consumption [32], and diet quality [33] were all based on self-report. Prevalent diseases are not included as covariates as it appears more likely that these were potential mediators (being caused by lack of/lower level of physical activity), where adjustment could lead to overadjustment bias.

### Statistical analyses

Descriptive statistics were used to compare demographic characteristics between the high and low physical activity groups (binary variable) for an easier interpretation. Higher PA level (> median) was used as the reference in the binary analyses to ensure correct estimation of additive interaction [34].

Cox proportional hazard regression models were used to examine the associations of PA and air pollution with health outcomes. Hazard ratios (HRs) and 95% confidence intervals (CI) were used to estimate the effect sizes. Follow-up started at the UK Biobank baseline assessment visit for self-reported PA and at the final date of accelerometer wear for objectively measured PA. Proportional hazard assumptions were checked via tests of the Schoenfeld residuals. None of the tests showed a significant violation of the assumptions.

Both multiplicative and additive interactions (relative excess risk due to additive interaction [RERI]) were calculated for the binary PA and air pollution variables. The above analyses also were replicated treating PA and air pollution variables as numeric variables (scaled to their sample interquartile range [IQR]) since even though dichotomised variables could be easier to interpret they could lead to imprecise and underpowered estimation.

The inferential analyses described above were conducted in the 10 imputed datasets using full conditional specification multiple imputation using chained equations to address missing data in covariates [35]. Rubin’s rule was used to pool the findings from the 10 imputed data. Ten imputations were selected based on the 16% total missingness in the data [36]. PA and air pollution variables were dichotomised as above or below the sample median. Complete case analysis including all people with non-missing data was conducted as a sensitivity analysis. Analysis of self-reported PA was replicated among people with accelerometer data only. Statistical analyses were performed using R statistical software 4.3.0 with the packages *mice*, *survival*, and *interactionR*.

## Results

Of the 502,536 UK Biobank participants, 414,644 were eligible for this study (Additional file 1: Fig. S1). People included in this study were, on average, 2 years younger, less deprived, and had slightly more (1 MET-h/week self-report and 2 MET-h/week accelerometer-measured) PA (Additional file 1: Table S1). Of these, 86,838 had valid accelerometer-measured PA. During the study period, 31,765 (7.7%) died, 70,299 (17.0%) had incident cancer, 25,130 (8.5%) developed type 2 diabetes, 33,284 (8.0%) had MACE, and 18,844 (4.5%) developed COPD. In the total sample, the median self-reported PA physical activity was 26.0 MET-hours/week, and the median accelerometer-measured PA physical activity was 61.1 MET-hours/week. Participants who had higher PA were on average less deprived, had lower exposure to air pollution, consumed more alcohol, had better diets, and were less likely to smoke (Table 1).

**Table 1:** Characteristics of participants

	**All participants**	**Self-reported PA** ** < median**	**Self-reported PA** ** ≥ median**	**Accelerometer-measured PA** ** < median**	**Accelerometer-measured PA** ** ≥ median**
	*N* = 414,644	*N* = 207,322	*N* = 207,322	*N* = 43,433	*N* = 43,405
Median (IQR) ages (years)	57.00 (50.00–63.00)	57.00 (50.00–63.00)	58.00 (50.00–63.00)	59.00 (52.00–63.00)	55.00 (48.00–61.00)
Sex					
Female	225,507 (54.4%)	117,568 (56.7%)	107,939 (52.1%)	23,909 (55%)	24,954 (57.5%)
Male	189,137 (45.6%)	89,754 (43.3%)	99,383 (47.9%)	19,524 (45.0%)	18,451 (42.5%)
Ethnicity					
White	389,850 (94.0%)	193,420 (93.3%)	196,430 (94.7%)	41,905 (96.5%)	41,755 (96.2%)
Black	7057 (1.7%)	3819 (1.8%)	3238 (1.6%)	336 (0.8%)	456 (1.1%)
South Asian	8507 (2.1%)	5113 (2.5%)	3394 (1.6%)	465 (1.1%)	437 (1.0%)
Chinese	1340 (0.3%)	754 (0.4%)	586 (0.3%)	85 (0.2%)	129 (0.3%)
Others/mixed	6421 (1.5%)	3437 (1.7%)	2984 (1.4%)	484 (1.1%)	519 (1.2%)
Median (IQR) deprivation index	− 2.20 (− 3.67, 0.39)	− 2.19 (− 3.67, 0.46)	− 2.22 (− 3.66, 0.31)	− 2.39 (− 3.78, − 0.04)	− 2.50 (− 3.83, − 0.32)
Smoking status					
Never	228,817 (55.2%)	115,166 (55.5%)	113,651 (54.8%)	24,209 (55.7%)	25,420 (58.6%)
Previous	142,123 (34.3%)	69,244 (33.4%)	72,879 (35.2%)	15,824 (36.4%)	15,176 (35.0%)
Current	42,293 (10.2%)	22,111 (10.7%)	20,182 (9.7%)	3298 (7.6%)	2733 (6.3%)
Median (IQR) lifestyle factors					
Alcohol drinking (units/week)	10.50 (2.76–22.5)	9.90 (1.50–21.00)	12.00 (3.00–24.00)	10.80 (3.00–21.75)	12.00 (4.50–21.75)
Diet quality	4.00 (3.00–6.00)	4.00 (3.00–5.00)	5.00 (3.00–6.00)	5.00 (3.00–6.00)	5.00 (4.00–6.00)
Self-reported PA (MET-h/week)	25.96 (10.95–55.40)	10.95 (4.65–17.58)	55.4 (37.55–89.30)	22.96 (9.90–45.66)	31.87 (15.23–60.95)
Accelerometer-measured PA (MET-h/week)	61.10 (46.00–79.20)	57.23 (42.98–74.11)	65.04 (49.29–83.91)	45.98 (36.32–53.59)	79.23 (69.18–94.73)
Median (IQR) air pollution level					
PM_2.5_ absorbance	1.13 (1.00–1.30)	1.13 (1.00–1.31)	1.12 (0.99–1.30)	1.12 (0.99–1.31)	1.11 (0.98–1.29)
PM_2.5_	9.93 (9.28–10.56)	9.94 (9.31–10.57)	9.91 (9.25–10.55)	9.88 (9.24–10.51)	9.83 (9.16–10.46)
PM_coarse_	6.11 (5.84–6.64)	6.11 (5.84–6.64)	6.10 (5.84–6.63)	6.10 (5.84–6.63)	6.09 (5.83–6.60)
PM_10_	16.02 (15.24–17.01)	16.04 (15.28–17.02)	16.01 (15.21–16.99)	16.02 (15.21–17.00)	15.98 (15.13–16.94)
NO_2_	26.07 (21.31–31.18)	26.23 (21.51–31.31)	25.91 (21.11–31.05)	25.74 (20.91–31.06)	25.29 (20.35–30.65)
NO_x_	42.13 (34.09–50.67)	42.35 (34.40–50.81)	41.90 (33.77–50.52)	41.46 (33.45–50.06)	40.83 (32.67–49.41)

Numbers shown are *n* (%) unless otherwise specified. *IQR* interquartile range

### Associations between air pollution and outcomes

**Fig. 1:**
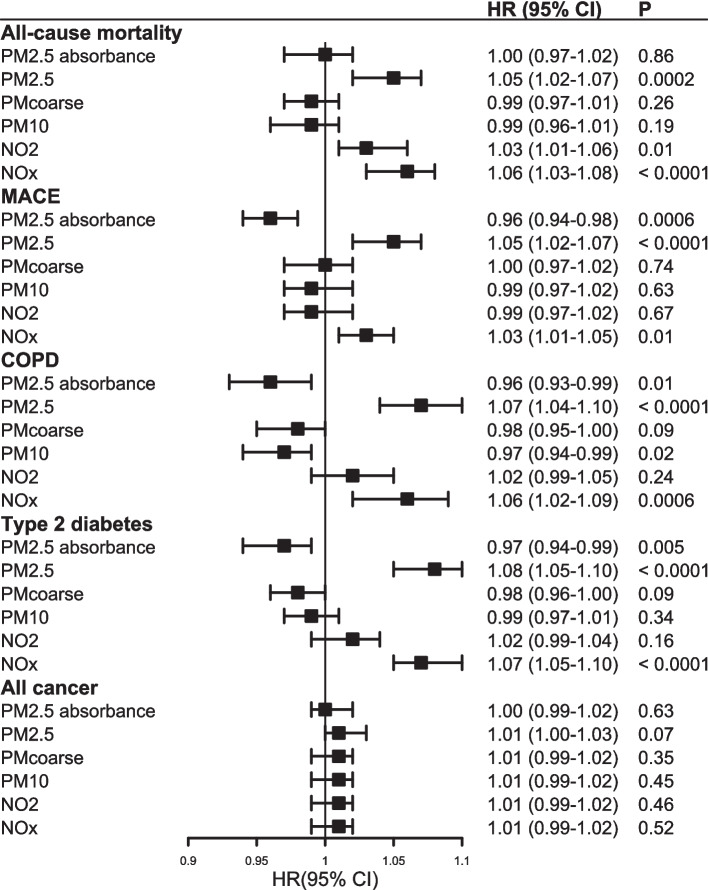
Association of air pollution level with health outcomes

Higher levels of PM_2.5_, but not PM_10_ or PM_coarse_, were associated with all outcomes except for cancer (Fig. 1; Additional file 1: Table S2). The strongest association was with COPD (HR_binary_ 1.07, 95% CI 1.04–1.10; HR_IQR_ 1.05; 95% CI 1.03–1.07). NO_x_, but not NO_2_, was associated with higher risk for all outcomes, except for cancer, and the strongest association was with T2D (HR_binary_ 1.07, 95% CI 1.05–1.10; HR_IQR_ 1.02; 95% CI 1.01–1.04). PM_2.5_ absorbance was associated with lower risk of all outcomes except for all-cause mortality and cancer.

Higher air pollution was consistently associated with lower PA level, except for PM_coarse_ and accelerometer-measured PA (Additional file 1: Table S3). Strongest association was observed in NO_2_ and accelerometer-measured PA. Each IQR increase in NO_2_ was associated with 1.64 MET-hours/week lower in PA.

### Associations between physical activity and outcomes

**Fig. 2:**
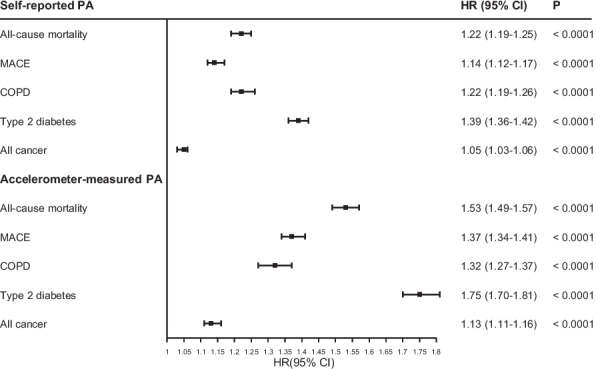
Association of physical activity level and health outcomes

Regardless of type of measurement or parameterisation, lower PA was associated with higher risk of health outcomes (Fig. 2; Additional file 1: Table S4). Generally, device-measured PA had a stronger association than self-reported PA; e.g. all-cause mortality HR_ACC_ 1.41 versus HR_SR_ 1.10 per IQR increase in PA. Of all health outcomes, the strongest association with accelerometer-measured PA was T2D (HR_ACC_ 1.55; 95% CI 1.52–1.58 per IQR increase in PA).

### Associations between physical activity and outcomes by air pollution level

**Fig. 3:**
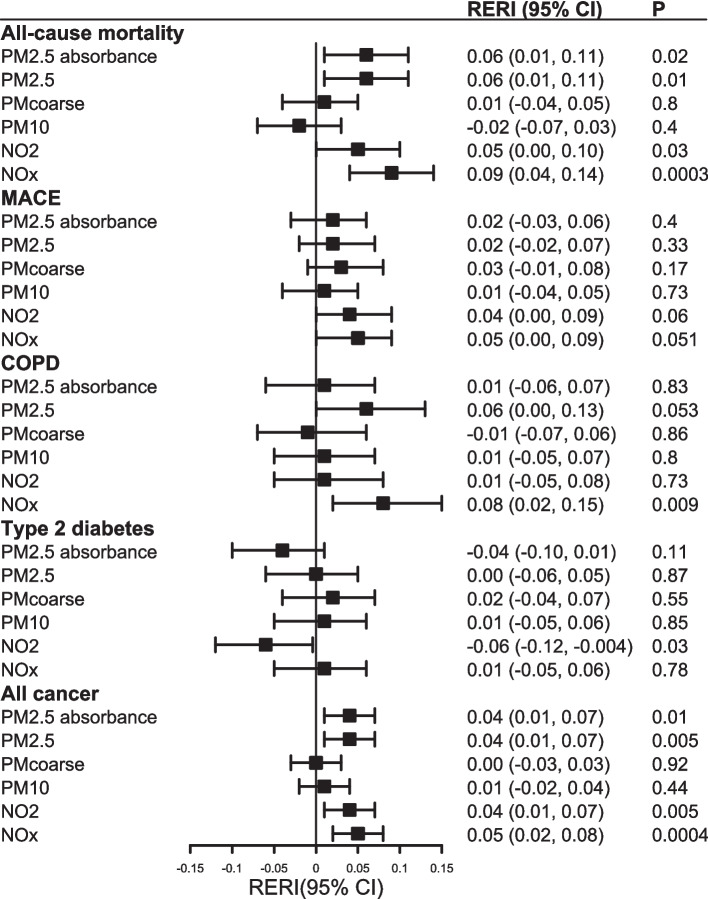
Additive interaction of lower self-reported physical activity and air pollution with health outcomes

**Fig. 4:**
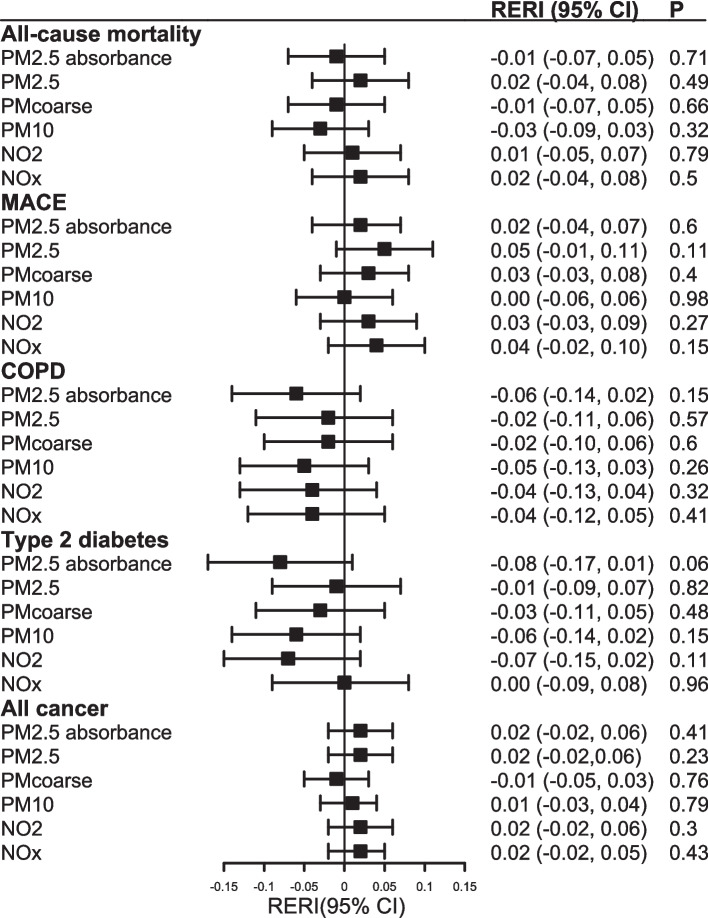
Additive interaction of lower accelerometer-measured physical activity and air pollution with health outcomes

The moderation role of air pollution on the association between self-reported PA and health outcomes is shown in Fig. 3 and Additional file 1: Table S5. Lower physical activity was associated with higher risk of health outcomes regardless of air pollution level. There was evidence of a positive, but weak, interaction between NO_x_ and self-reported PA in relation to all-cause mortality (RERI 0.09, 95% CI 0.04, 0.14), COPD (RERI 0.08, 95% CI 0.02, 0.15), and cancer (RERI 0.05, 95% CI 0.02, 0.08), indicating that the associations between PA and these outcomes were stronger in areas with higher NO_x_ levels. In contrast, there was evidence of a negative, but weak, interaction between NO_2_ and PA in relation to T2D (RERI − 0.06, 95% CI − 0.12, − 0.004), suggesting the association between PA and T2D was slightly weaker in areas with higher NO_2_ levels. However, none of these interactions was replicated when PA was measured using accelerometers (Fig. 4 and Additional file 1: Table S6). Complete case analyses (Additional file 1: Tables S7 and S8), analysis using continuous variables (Additional file 1: Tables S9 and S10), and analysis of self-reported PA variables among people with accelerometer data (Additional file 1: Table S11) showed similar results.

## Discussion

### Key results

This study systematically explored whether air pollution modified the associations between PA and four relevant long-term conditions, as well as all-cause mortality. Overall, the associations between PA and health were much stronger than those between air pollution and health, suggesting the benefits from PA could outweigh the harms from air pollution. Furthermore, we found no strong evidence to suggest that, within the UK, the potential health benefits of PA were lower in areas with higher air pollution, even though there were consistent associations between air pollution and PA level.

### Interpretation

The strongest association for PM_2.5_ was with COPD which is consistent with existing research [37]. Exposure to PM_2.5_ can directly induce inflammation in the pulmonary system, which is a primary mechanism of COPD [38–40]. The association of PM_2.5_ with all-cause mortality and MACE has been reported in a large number of studies [41–43]. PM_2.5_ may contribute to cardiovascular events by activating oxidative stress and inflammatory pathways [44, 45]. Our study finding on NO_X_ and type 2 diabetes was also consistent with the literature [46].

This study found that higher levels of PA, whether self-reported or measured objectively, were associated with lower risk of health outcomes. The health benefits of PA have been replicated consistently [47–49]. We demonstrated stronger associations for objectively measured PA which verified the presence of regression dilution bias when self-reported PA is used. This difference between self-reported and accelerometer-based measures of PA may be explained by the fact that accelerometers can measure body activity more accurately and avoid recall or reporting bias [50]. Of all the long-term conditions included in the study, PA had the strongest association with type 2 diabetes. This is consistent with the findings of Coogan et al. [51].

We found little evidence to support any meaningful negative interaction between PA level and air pollution in relation to health outcomes. This finding is consistent with previous epidemiological studies conducted in Denmark where air pollution is generally lower [12–14]. However, it contrasts with a South Korean study which found that increasing PA over time was associated with higher CVD risk in area with high air pollution [52]. It should, however, be noted that the South Korean study defined higher air pollution as annual PM_2.5_ > 26.4 µg/m^3^, exceeding the maximum exposure of 21.3 µg/m^3^ estimated in our study. Interestingly, a randomised controlled trial showed that a 2-h walking exercise resulted in immediate improvement in lung function but only if the exercise was done in a lower air pollution area [53]. The moderation role of air pollution may also be different in younger people. There have been studies that showed interactions between PA and air pollution in relation to asthma [10] and cardiorespiratory fitness [11] in children. There was some evidence for positive interaction between air pollution and self-reported PA, where people in higher air pollution areas could be particularly susceptible to the health risk due to low PA. However, since these findings were not replicable in objectively measured PA, it could simply be measurement biases associated with socioeconomic background (since socioeconomic background is also associated with air pollution), or spurious findings due to multiple testing.

### Strengths and limitations

A strength of this study was the use of both self-reported and accelerometer-measured physical activity, a series of validated air pollution measures and multiple outcome measures. The large sample size provided sufficient statistical power to detect any meaningful interactions (± 7% for multiplicative and ± 8% additive interaction). However, this study also has some limitations. Firstly, since UK Biobank only provides participants’ residential address at baseline, the estimation of exposure to air pollution was based on exposure at home at baseline and did not take account of subsequent relocation or exposures at other places, such as work, and indoor air pollution for individuals who take part in indoor physical activity. However, it would not have been feasible to have personalised air pollution monitor for a population cohort of this size, which is essential for statistical power for rarer health outcomes. There are now air pollution indices from newer models, e.g. those from DEFRA, which in theory could provide time-varying estimates, but it is currently not available routinely in UK Biobank. Furthermore, air pollution and physical activity were not measured at the same time. Both of these measurement issues would likely to result in non-differential misclassification which could underestimate the associations. Secondly, despite the sample size, the numbers of site-specific cancers in UK Biobank, especially for those with accelerometer data, are small, limiting us from examining the associations for those outcomes. Thirdly, as with all observational studies, associations cannot be assumed to be causal and, in spite of adjusting for a range of confounders, residual confounding cannot be ruled out. This is particularly the case as we decided not to adjust for prevalent diseases to avoid overadjustment bias, even though prevalent diseases could lead to less physical activity. Future studies with repeated measures of physical activity could potentially explore such relationship. Fourthly, the study included air pollutants agnostically and therefore conducted a larger number of tests. Inflation of family-wise type 1 error is possible and could explain some of the statistically significant findings. Fifthly, UK Biobank has a healthy volunteer bias and some findings might not be generalisable to the UK population. Sixthly, due to computational difficulties we were not able to conduct more imputations, which could affect the power to detect associations and interactions. Lastly, all data in this study were based on the UK. The findings of this study may not be applicable to other regions with different climates and different levels or distribution of air pollution.

## Conclusions

This study showed that, in the UK, PA levels were consistently associated with lower risk of long-term conditions and mortality, even in areas with higher air pollution levels. Whilst air pollution is harmful to health and should be reduced, PA should be promoted regardless of air pollution levels.

## Supplementary Information

Additional file 1: Figure S1; Tables S1–S11. Fig. S1 Participant flowchart. Table S1 Comparison of UK Biobank participant characteristics by inclusion. Table S2 Association between air pollution and physical activity level. Table S3 Association of air pollution level with health outcomes. Table S4 Association of physical activity level and health outcomes. Table S5 Association between lower self-reported physical activity level and health outcomes by air pollution level. Table S6 Association between lower accelerometer-measured physical activity level and health outcomes by air pollution level. Table S7 Association between self-reported lower physical activity level and health outcomes by air pollution level in complete case analysis. Table S8 Association between accelerometer-measured lower physical activity level. Table S9 Association between per IQR decrease in self-reported physical activity level and health outcomes by air pollution level. Table S10 Association between per IQR decrease in accelerometer-measured physical activity level and health outcomes by air pollution level. Table S11 Association between lower self-reported physical activity level and health outcomes by air pollution level among people with valid accelerometer data.

## Data Availability

Request of data can be made to the UK Biobank ( [https://www.ukbiobank.ac.uk/](https://www.ukbiobank.ac.uk) ). R codes for this paper can be found on Github ( [https://github.com/fredho-42/PA-AQL-BMJMed2025](https://github.com/fredho-42/PA-AQL-BMJMed2025) ).

## Abbreviations

COPD: Chronic obstructive pulmonary disease
IPAQ: International Physical Activity Questionnaire
HR: Hazard ratio
IQR: Interquartile range
LPA: Light intensity physical activity
MACE: Major adverse cardiovascular events
MPA: Moderate intensity physical activity
MET: Metabolic equivalent of task
NO_x_: Nitrogen oxides
NO_2_: Nitrogen dioxide
PA: Physical activity
PM_2.5_: Particulate matters less than 2.5 µm in diameter
PM_10_: Particulate matters less than 10 µm in diameter
PM_coarse_: Particulate matters between 2.5 and 10 µm in diameter
RERI: Relative excess risk of additive interaction
T2D: Type 2 diabetes
VPA: Vigorous intensity physical activity
