# Supplementary material for: Physical activity, air pollution, and incident long-term conditions: a prospective cohort study
Source: BMC Med. 2025 Aug 22;23:491. doi: 10.1186/s12916-025-04338-x (PMC12374305; doi:10.1186/s12916-025-04338-x)
Supplement: Supplementary file 1 — Additional file 1: Figure S1; Tables S1–S11. Fig. S1 Participant flowchart. Table S1 Comparison of UK Biobank participant characteristics by inclusion. Table S2 Association between air pollution and physical activity level. Table S3 Association of air pollution level with health outcomes. Table S4 Association of physical activity level and health outcomes. Table S5 Association between lower self-reported physical activity level and health outcomes by air pollution level. Table S6 Association between lower accelerometer-measured physical activity level and health outcomes by air pollution level. Table S7 Association between self-reported lower physical activity level and health outcomes by air pollution level in complete case analysis. Table S8 Association between accelerometer-measured lower physical activity level. Table S9 Association between per IQR decrease in self-reported physical activity level and health outcomes by air pollution level. Table S10 Association between per IQR decrease in accelerometer-measured physical activity level and health outcomes by air pollution level. Table S11 Association between lower self-reported physical activity level and health outcomes by air pollution level among people with valid accelerometer data. [file 12916_2025_4338_MOESM1_ESM.docx]

**Additional File 1**

Figure S1. Participant flowchart

502,366 UK Biobank participants

N = 481,492

20,874 excluded due to missing self-reported PA data

N = 441,661

39,831 excluded with no valid air pollution data

N = 441,644 included in the self-reported PA analysis

17 excluded who had the included long-term conditions before baseline

Table S1. Comparison of UK Biobank participants characteristics by inclusion

|  | **Excluded** | **Included** | **p-value** |
| --- | --- | --- | --- |
| Number | 87,722 | 414,644 |  |
| Median (IQR) ages (years) | 59 (52, 64) | 57 (50, 63) | <0.001 |
| Sex |  |  | 0.60 |
| Female | 47,782 (54%) | 225,507 (54%) |  |
| Male | 39,924 (46%) | 189,137 (46%) |  |
| Ethnicity |  |  | <0.001 |
| White | 82,706 (96%) | 389,850 (94%) |  |
| Black | 1,001 (1.2%) | 7,057 (1.7%) |  |
| South Asian | 1,372 (1.6%) | 8,507 (2.1%) |  |
| Chinese | 233 (0.3%) | 1,340 (0.3%) |  |
| Others/Mixed | 1087 (1.3%) | 6,421 (1.5%) |  |
| Median (IQR) deprivation index | -1.78 (-3.53, 1.37) | -2.20 (-3.67, 0.39) | <0.001 |
| Smoking status |  |  | <0.001 |
| Never | 44,631 (52%) | 228,817 (55%) |  |
| Previous | 30,885 (36%) | 142,123 (34%) |  |
| Current | 10,668 (12%) | 42,293 (10%) |  |
| Median (IQR) lifestyle factors |  |  |  |
| Alcohol drinking (units/wk) | 10 (2, 21) | 11 (3, 23) | <0.001 |
| Diet quality | 4.00 (3.00, 5.00) | 4.00 (3.00, 6.00) | <0.001 |
| Self-reported PA (MET-h/wk) | 25 (10, 52) | 26 (11, 55) | <0.001 |
| Accelerometer-measured PA (MET-h/wk) | 59 (43, 77) | 61 (46, 79) | <0.001 |

Numbers shown are n (%) unless otherwise specified. IQR: Interquartile range. P-values calculated from Wilcoxon rank sum test for numeric variables and Pearson's Chi-squared test for categorical variables.

Table S2. Association between air pollution and physical activity level

|  | **Self-reported** | | **Accelerometer-measured** | |
| --- | --- | --- | --- | --- |
|  | **Beta (95% CI)** | **P** | **Beta (95% CI)** | **P** |
| PM_2.5_ absorbance | -0.84 (-1.11, -0.56) | <0.0001 | -1.06 (-1.21, -0.91) | <0.0001 |
| PM_2.5_ | -1.28 (-1.57, -0.99) | <0.0001 | -1.13 (-1.3, -0.96) | <0.0001 |
| PM_course_ | -0.14 (-0.26, -0.03) | 0.01 | -0.07 (-0.18, 0.04) | 0.23 |
| PM_10_ | -0.56 (-0.69, -0.42) | <0.0001 | -0.64 (-0.76, -0.52) | <0.0001 |
| NO_2_ | -1.43 (-1.74, -1.13) | <0.0001 | -1.64 (-1.82, -1.45) | <0.0001 |
| NO_x_ | -0.93 (-1.16, -0.7) | <0.0001 | -0.81 (-0.96, -0.66) | <0.0001 |

Analyses adjusted for age, sex, ethnicity, deprivation index, smoking status, alcohol consumption, diet quality.

Table S3. Association of air pollution level with health outcomes

|  | **Below median** | | **Per IQR increase** | |
| --- | --- | --- | --- | --- |
|  | **HR (95% CI)** | **P** | **HR (95% CI)** | **P** |
| **All-cause mortality** |  |  |  |  |
| PM_2.5_ absorbance | 1.00 (0.97-1.02) | 0.86 | 0.98 (0.97-0.99) | 0.002 |
| PM_2.5_ | 1.05 (1.02-1.07) | 0.0002 | 1.03 (1.01-1.04) | 0.0002 |
| PM_coarse_ | 0.99 (0.97-1.01) | 0.26 | 0.99 (0.98-1.00) | 0.12 |
| PM_10_ | 0.99 (0.96-1.01) | 0.19 | 1.00 (0.99-1.01) | 0.78 |
| NO_2_ | 1.03 (1.01-1.06) | 0.01 | 1.01 (0.99-1.03) | 0.27 |
| NO_x_ | 1.06 (1.03-1.08) | < 0.0001 | 1.02 (1.01-1.03) | 0.002 |
| **MACE** |  |  |  |  |
| PM_2.5_ absorbance | 0.96 (0.94-0.98) | 0.0006 | 0.96 (0.95-0.97) | < 0.0001 |
| PM_2.5_ | 1.05 (1.02-1.07) | < 0.0001 | 1.02 (1.01-1.04) | 0.002 |
| PM_coarse_ | 1.00 (0.97-1.02) | 0.74 | 1.00 (0.99-1.01) | 0.54 |
| PM_10_ | 0.99 (0.97-1.02) | 0.63 | 1.01 (1.00-1.02) | 0.17 |
| NO_2_ | 0.99 (0.97-1.02) | 0.67 | 0.99 (0.97-1.00) | 0.07 |
| NO_x_ | 1.03 (1.01-1.05) | 0.01 | 1.01 (1.00-1.02) | 0.054 |
| **COPD** |  |  |  |  |
| PM_2.5_ absorbance | 0.96 (0.93-0.99) | 0.01 | 0.95 (0.93-0.97) | < 0.0001 |
| PM_2.5_ | 1.07 (1.04-1.10) | < 0.0001 | 1.05 (1.03-1.07) | < 0.0001 |
| PM_coarse_ | 0.98 (0.95-1.00) | 0.09 | 0.99 (0.98-1.00) | 0.14 |
| PM_10_ | 0.97 (0.94-0.99) | 0.02 | 1.00 (0.99-1.02) | 0.56 |
| NO_2_ | 1.02 (0.99-1.05) | 0.24 | 1.00 (0.98-1.02) | 0.92 |
| NO_x_ | 1.06 (1.02-1.09) | 0.0006 | 1.02 (1.01-1.04) | 0.004 |
| **Type 2 diabetes** |  |  |  |  |
| PM_2.5_ absorbance | 0.97 (0.94-0.99) | 0.005 | 0.97 (0.96-0.98) | < 0.0001 |
| PM_2.5_ | 1.08 (1.05-1.10) | < 0.0001 | 1.04 (1.03-1.06) | < 0.0001 |
| PM_coarse_ | 0.98 (0.96-1.00) | 0.09 | 1.00 (0.99-1.01) | 0.38 |
| PM_10_ | 0.99 (0.97-1.01) | 0.34 | 1.02 (1.01-1.03) | 0.001 |
| NO_2_ | 1.02 (0.99-1.04) | 0.16 | 1.00 (0.98-1.01) | 0.81 |
| NO_x_ | 1.07 (1.05-1.10) | < 0.0001 | 1.02 (1.01-1.04) | 0.0002 |
| **All cancer** |  |  |  |  |
| PM_2.5_ absorbance | 1.00 (0.99-1.02) | 0.63 | 1.00 (0.99-1.01) | 0.94 |
| PM_2.5_ | 1.01 (1.00-1.03) | 0.07 | 1.01 (1.00-1.02) | 0.07 |
| PM_coarse_ | 1.01 (0.99-1.02) | 0.35 | 1.00 (1.00-1.01) | 0.50 |
| PM_10_ | 1.01 (0.99-1.02) | 0.45 | 1.01 (1.00-1.01) | 0.07 |
| NO_2_ | 1.01 (0.99-1.02) | 0.46 | 1.01 (1.00-1.02) | 0.27 |
| NO_x_ | 1.01 (0.99-1.02) | 0.52 | 1.00 (1.00-1.01) | 0.29 |

Analyses adjusted for age, sex, ethnicity, deprivation index, smoking status, alcohol consumption, diet quality.

Table S4. Association of physical activity level and health outcomes

|  | **Self-reported** | | **Accelerometer-measured** | |
| --- | --- | --- | --- | --- |
|  | **HR (95% CI)** | **P value** | **HR (95% CI)** | **P value** |
| All-cause mortality |  |  |  |  |
| Below median | 1.22 (1.19-1.25) | < 0.0001 | 1.53 (1.49-1.57) | < 0.0001 |
| Per IQR decrease | 1.10 (1.08-1.11) | < 0.0001 | 1.41 (1.39-1.43) | < 0.0001 |
| MACE |  |  |  |  |
| Below median | 1.14 (1.12-1.17) | < 0.0001 | 1.37 (1.34-1.41) | < 0.0001 |
| Per IQR decrease | 1.05 (1.04-1.07) | < 0.0001 | 1.28 (1.26-1.31) | < 0.0001 |
| COPD |  |  |  |  |
| Below median | 1.22 (1.19-1.26) | < 0.0001 | 1.32 (1.27-1.37) | < 0.0001 |
| Per IQR decrease | 1.07 (1.05-1.08) | < 0.0001 | 1.23 (1.20-1.26) | < 0.0001 |
| Type 2 diabetes |  |  |  |  |
| Below median | 1.39 (1.36-1.42) | < 0.0001 | 1.75 (1.70-1.81) | < 0.0001 |
| Per IQR decrease | 1.18 (1.16-1.20) | < 0.0001 | 1.55 (1.52-1.58) | < 0.0001 |
| All cancer |  |  |  |  |
| Below median | 1.05 (1.03-1.06) | < 0.0001 | 1.13 (1.11-1.16) | < 0.0001 |
| Per IQR decrease | 1.03 (1.02-1.03) | < 0.0001 | 1.09 (1.08-1.11) | < 0.0001 |

Analyses adjusted for age, sex, ethnicity, deprivation index, smoking status, alcohol consumption, diet quality.

Table S5. Association between lower self-reported physical activity level and health outcomes by air pollution level

|  | **HR stratified by air pollution level** | | **Interaction** | | | |
| --- | --- | --- | --- | --- | --- | --- |
|  | **Lower** | **Higher** | **HR_higher_ / HR_lower_** | **P** | **RERI** | **P** |
| **All-cause mortality** |  |  |  |  |  |  |
| **PM_2.5_ absorbance** | **1.19 (1.15-1.23)** | **1.25 (1.22-1.30)** | **1.06 (1.01-1.10)** | **0.02** | **0.06 (0.01, 0.11)** | **0.02** |
| **PM_2.5_** | **1.19 (1.15-1.23)** | **1.25 (1.21-1.29)** | **1.05 (1.00-1.10)** | **0.04** | **0.06 (0.01, 0.11)** | **0.01** |
| PM_coarse_ | 1.22 (1.18-1.25) | 1.23 (1.19-1.27) | 1.01 (0.97-1.05) | 0.70 | 0.01 (-0.04, 0.05) | 0.80 |
| PM_10_ | 1.23 (1.19-1.27) | 1.21 (1.17-1.25) | 0.98 (0.94-1.03) | 0.49 | -0.02 (-0.07, 0.03) | 0.40 |
| **NO_2_** | **1.19 (1.16-1.23)** | **1.25 (1.21-1.28)** | **1.04 (1.00-1.09)** | **0.06** | **0.05 (0.00, 0.10)** | **0.03** |
| **NO_x_** | **1.18 (1.14-1.21)** | **1.26 (1.22-1.30)** | **1.07 (1.03-1.12)** | **0.002** | **0.09 (0.04, 0.14)** | **0.0003** |
| **MACE** |  |  |  |  |  |  |
| PM_2.5_ absorbance | 1.13 (1.10-1.16) | 1.16 (1.12-1.19) | 1.02 (0.98-1.07) | 0.27 | 0.02 (-0.03, 0.06) | 0.40 |
| PM_2.5_ | 1.13 (1.10-1.17) | 1.15 (1.12-1.19) | 1.02 (0.97-1.06) | 0.49 | 0.02 (-0.02, 0.07) | 0.33 |
| PM_coarse_ | 1.13 (1.09-1.16) | 1.16 (1.13-1.20) | 1.03 (0.99-1.08) | 0.16 | 0.03 (-0.01, 0.08) | 0.17 |
| PM_10_ | 1.14 (1.10-1.17) | 1.15 (1.11-1.18) | 1.01 (0.97-1.05) | 0.70 | 0.01 (-0.04, 0.05) | 0.73 |
| NO_2_ | 1.12 (1.08-1.15) | 1.17 (1.13-1.20) | 1.04 (1.00-1.09) | 0.052 | 0.04 (0.00, 0.09) | 0.06 |
| NO_x_ | 1.12 (1.09-1.16) | 1.16 (1.13-1.20) | 1.04 (1.00-1.09) | 0.08 | 0.05 (0.00, 0.09) | 0.051 |
| **COPD** |  |  |  |  |  |  |
| PM_2.5_ absorbance | 1.21 (1.16-1.27) | 1.23 (1.18-1.28) | 1.01 (0.96-1.07) | 0.62 | 0.01 (-0.06, 0.07) | 0.83 |
| PM_2.5_ | 1.19 (1.14-1.25) | 1.24 (1.20-1.29) | 1.04 (0.99-1.11) | 0.14 | 0.06 (0.00, 0.13) | 0.053 |
| PM_coarse_ | 1.22 (1.17-1.27) | 1.22 (1.18-1.27) | 1.00 (0.94-1.06) | 0.99 | -0.01 (-0.07, 0.06) | 0.86 |
| PM_10_ | 1.21 (1.16-1.27) | 1.23 (1.18-1.28) | 1.02 (0.96-1.08) | 0.61 | 0.01 (-0.05, 0.07) | 0.80 |
| NO_2_ | 1.22 (1.17-1.27) | 1.23 (1.18-1.27) | 1.01 (0.95-1.07) | 0.81 | 0.01 (-0.05, 0.08) | 0.73 |
| NO_x_ | **1.18 (1.13-1.23)** | **1.26 (1.21-1.31)** | **1.07 (1.01-1.13)** | **0.03** | **0.08 (0.02, 0.15)** | **0.009** |
| **Type 2 diabetes** |  |  |  |  |  |  |
| PM_2.5_ absorbance | 1.41 (1.37-1.46) | 1.38 (1.34-1.42) | 0.98 (0.93-1.02) | 0.30 | -0.04 (-0.10, 0.01) | 0.11 |
| PM_2.5_ | 1.41 (1.37-1.46) | 1.37 (1.33-1.42) | 0.97 (0.93-1.02) | 0.24 | 0.00 (-0.06, 0.05) | 0.87 |
| PM_coarse_ | 1.38 (1.33-1.42) | 1.41 (1.36-1.45) | 1.02 (0.98-1.07) | 0.35 | 0.02 (-0.04, 0.07) | 0.55 |
| PM_10_ | 1.39 (1.34-1.43) | 1.40 (1.36-1.44) | 1.01 (0.96-1.06) | 0.69 | 0.01 (-0.05, 0.06) | 0.85 |
| **NO_2_** | **1.44 (1.39-1.49)** | **1.36 (1.32-1.40)** | **0.95 (0.90-0.99)** | **0.02** | **-0.06 (-0.12, -0.004)** | **0.03** |
| NO_x_ | 1.40 (1.36-1.45) | 1.38 (1.34-1.42) | 0.98 (0.94-1.03) | 0.50 | 0.01 (-0.05, 0.06) | 0.78 |
| **All cancer** |  |  |  |  |  |  |
| **PM_2.5_ absorbance** | **1.03 (1.01-1.05)** | **1.07 (1.04-1.09)** | **1.04 (1.01-1.07)** | **0.01** | **0.04 (0.01, 0.07)** | **0.01** |
| **PM_2.5_** | **1.02 (1.00-1.05)** | **1.07 (1.05-1.09)** | **1.04 (1.01-1.07)** | **0.006** | **0.04 (0.01, 0.07)** | **0.005** |
| PM_coarse_ | 1.05 (1.02-1.07) | 1.04 (1.02-1.07) | 1.00 (0.97-1.03) | 0.91 | 0.00 (-0.03, 0.03) | 0.92 |
| PM_10_ | 1.04 (1.02-1.06) | 1.05 (1.03-1.07) | 1.01 (0.98-1.04) | 0.45 | 0.01 (-0.02, 0.04) | 0.44 |
| **NO_2_** | **1.02 (1.00-1.05)** | **1.07 (1.05-1.09)** | **1.04 (1.01-1.07)** | **0.006** | **0.04 (0.01, 0.07)** | **0.005** |
| **NO_x_** | **1.02 (1.00-1.04)** | **1.07 (1.05-1.10)** | **1.05 (1.02-1.09)** | **0.0004** | **0.05 (0.02, 0.08)** | **0.0004** |

Analyses adjusted for age, sex, ethnicity, deprivation index, smoking status, alcohol consumption, diet quality.

Table S6. Association between lower accelerometer-measured physical activity level and health outcomes by air pollution level

|  | **HR stratified by air pollution level** | | **Interaction** | | | |
| --- | --- | --- | --- | --- | --- | --- |
|  | **Lower** | **Higher** | **HR_higher_ / HR_lower_** | **P** | **RERI** | **P** |
| **All-cause mortality** |  |  |  |  |  |  |
| PM_2.5_ absorbance | 1.53 (1.48-1.59) | 1.53 (1.48-1.59) | 1.00 (0.95-1.05) | 0.97 | -0.01 (-0.07, 0.05) | 0.71 |
| PM_2.5_ | 1.53 (1.48-1.59) | 1.53 (1.47-1.58) | 1.00 (0.95-1.05) | 0.86 | 0.02 (-0.04, 0.08) | 0.49 |
| PM_coarse_ | 1.53 (1.47-1.58) | 1.54 (1.48-1.60) | 1.01 (0.96-1.06) | 0.78 | -0.01 (-0.07, 0.05) | 0.66 |
| PM_10_ | 1.54 (1.49-1.60) | 1.53 (1.47-1.58) | 0.99 (0.94-1.04) | 0.77 | -0.03 (-0.09, 0.03) | 0.32 |
| NO_2_ | 1.53 (1.47-1.59) | 1.54 (1.48-1.59) | 1.00 (0.96-1.06) | 0.85 | 0.01 (-0.05, 0.07) | 0.79 |
| NO_x_ | 1.53 (1.48-1.59) | 1.53 (1.48-1.59) | 1.00 (0.95-1.05) | 0.98 | 0.02 (-0.04, 0.08) | 0.50 |
| **MACE** |  |  |  |  |  |  |
| PM_2.5_ absorbance | 1.35 (1.30-1.40) | 1.39 (1.34-1.45) | 1.03 (0.98-1.09) | 0.25 | 0.02 (-0.04, 0.07) | 0.60 |
| PM_2.5_ | 1.35 (1.30-1.40) | 1.39 (1.34-1.44) | 1.03 (0.97-1.08) | 0.35 | 0.05 (-0.01, 0.11) | 0.11 |
| PM_coarse_ | 1.35 (1.30-1.40) | 1.39 (1.34-1.44) | 1.03 (0.98-1.08) | 0.30 | 0.03 (-0.03, 0.08) | 0.40 |
| PM_10_ | 1.37 (1.32-1.42) | 1.38 (1.33-1.43) | 1.01 (0.95-1.06) | 0.83 | 0.00 (-0.06, 0.06) | 0.98 |
| NO_2_ | 1.35 (1.30-1.40) | 1.40 (1.35-1.45) | 1.04 (0.98-1.09) | 0.19 | 0.03 (-0.03, 0.09) | 0.27 |
| NO_x_ | 1.35 (1.30-1.40) | 1.39 (1.34-1.44) | 1.03 (0.98-1.09) | 0.24 | 0.04 (-0.02, 0.10) | 0.15 |
| **COPD** |  |  |  |  |  |  |
| PM_2.5_ absorbance | 1.35 (1.28-1.42) | 1.29 (1.23-1.36) | 0.96 (0.89-1.03) | 0.23 | -0.06 (-0.14, 0.02) | 0.15 |
| PM_2.5_ | 1.34 (1.27-1.42) | 1.29 (1.23-1.36) | 0.96 (0.90-1.03) | 0.30 | -0.02 (-0.11, 0.06) | 0.57 |
| PM_coarse_ | 1.33 (1.26-1.40) | 1.31 (1.25-1.38) | 0.99 (0.92-1.06) | 0.80 | -0.02 (-0.10, 0.06) | 0.60 |
| PM_10_ | 1.34 (1.27-1.41) | 1.30 (1.24-1.37) | 0.97 (0.90-1.04) | 0.42 | -0.05 (-0.13, 0.03) | 0.26 |
| NO_2_ | 1.35 (1.28-1.42) | 1.30 (1.23-1.36) | 0.96 (0.89-1.03) | 0.29 | -0.04 (-0.13, 0.04) | 0.32 |
| NO_x_ | 1.35 (1.28-1.43) | 1.29 (1.23-1.36) | 0.96 (0.89-1.03) | 0.23 | -0.04 (-0.12, 0.05) | 0.41 |
| **Type 2 diabetes** |  |  |  |  |  |  |
| PM_2.5_ absorbance | 1.78 (1.71-1.86) | 1.73 (1.66-1.81) | 0.97 (0.91-1.03) | 0.34 | -0.08 (-0.17, 0.01) | 0.06 |
| PM_2.5_ | 1.78 (1.70-1.87) | 1.72 (1.65-1.80) | 0.97 (0.91-1.03) | 0.28 | -0.01 (-0.09, 0.07) | 0.82 |
| PM_coarse_ | 1.76 (1.68-1.84) | 1.75 (1.67-1.82) | 0.99 (0.93-1.05) | 0.80 | -0.03 (-0.11, 0.05) | 0.48 |
| PM_10_ | 1.79 (1.71-1.87) | 1.72 (1.65-1.80) | 0.97 (0.91-1.03) | 0.26 | -0.06 (-0.14, 0.02) | 0.15 |
| NO_2_ | 1.80 (1.72-1.88) | 1.72 (1.65-1.79) | 0.95 (0.90-1.01) | 0.13 | -0.07 (-0.15, 0.02) | 0.11 |
| NO_x_ | 1.78 (1.70-1.86) | 1.73 (1.66-1.81) | 0.97 (0.92-1.04) | 0.41 | 0.00 (-0.09, 0.08) | 0.96 |
| **All cancer** |  |  |  |  |  |  |
| PM_2.5_ absorbance | 1.13 (1.10-1.15) | 1.14 (1.11-1.17) | 1.02 (0.98-1.05) | 0.43 | 0.02 (-0.02, 0.06) | 0.41 |
| PM_2.5_ | 1.12 (1.09-1.15) | 1.15 (1.11-1.18) | 1.02 (0.98-1.06) | 0.27 | 0.02 (-0.02,0.06) | 0.23 |
| PM_coarse_ | 1.14 (1.11-1.17) | 1.13 (1.10-1.16) | 0.99 (0.96-1.03) | 0.77 | -0.01 (-0.05, 0.03) | 0.76 |
| PM_10_ | 1.13 (1.10-1.16) | 1.14 (1.11-1.17) | 1.01 (0.97-1.04) | 0.79 | 0.01 (-0.03, 0.04) | 0.79 |
| NO_2_ | 1.12 (1.09-1.15) | 1.15 (1.12-1.18) | 1.02 (0.98-1.06) | 0.28 | 0.02 (-0.02, 0.06) | 0.30 |
| NO_x_ | 1.13 (1.10-1.15) | 1.14 (1.11-1.17) | 1.02 (0.98-1.05) | 0.42 | 0.02 (-0.02, 0.05) | 0.43 |

Analyses adjusted for age, sex, ethnicity, deprivation index, smoking status, alcohol consumption, diet quality.

Table S7. Association between self-reported lower physical activity level and health outcomes by air pollution level in complete case analysis

|  | **HR stratified by air pollution level** | | **Interaction** | | | |
| --- | --- | --- | --- | --- | --- | --- |
|  | **Lower** | **Higher** | **HR_higher_ / HR_lower_** | **P** | **RERI** | **P** |
| **All-cause mortality** |  |  |  |  |  |  |
| **PM_2.5_ absorbance** | **1.19 (1.15-1.23)** | **1.25 (1.22-1.30)** | **1.06 (1.01-1.10)** | **0.02** | **0.06 (0.01, 0.11)** | **0.02** |
| **PM_2.5_** | **1.19 (1.15-1.23)** | **1.25 (1.21-1.29)** | **1.05 (1.00-1.10)** | **0.04** | **0.06 (0.01, 0.11)** | **0.01** |
| PM_coarse_ | 1.22 (1.18-1.25) | 1.23 (1.19-1.27) | 1.01 (0.97-1.05) | 0.70 | 0.01 (-0.04, 0.05) | 0.80 |
| PM_10_ | 1.23 (1.19-1.27) | 1.21 (1.17-1.25) | 0.98 (0.94-1.03) | 0.49 | -0.02 (-0.07, 0.03) | 0.40 |
| **NO_2_** | **1.19 (1.16-1.23)** | **1.25 (1.21-1.28)** | **1.04 (1.00-1.09)** | **0.06** | **0.05 (0.00, 0.10)** | **0.03** |
| **NO_x_** | **1.18 (1.14-1.21)** | **1.26 (1.22-1.30)** | **1.07 (1.03-1.12)** | **0.002** | **0.09 (0.04, 0.14)** | **0.0003** |
| **MACE** |  |  |  |  |  |  |
| PM_2.5_ absorbance | 1.13 (1.10-1.16) | 1.16 (1.12-1.19) | 1.02 (0.98-1.07) | 0.27 | 0.02 (-0.03, 0.06) | 0.40 |
| PM_2.5_ | 1.13 (1.10-1.17) | 1.15 (1.12-1.19) | 1.02 (0.97-1.06) | 0.49 | 0.02 (-0.02, 0.07) | 0.33 |
| PM_coarse_ | 1.13 (1.09-1.16) | 1.16 (1.13-1.20) | 1.03 (0.99-1.08) | 0.16 | 0.03 (-0.01, 0.08) | 0.17 |
| PM_10_ | 1.14 (1.10-1.17) | 1.15 (1.11-1.18) | 1.01 (0.97-1.05) | 0.70 | 0.01 (-0.04, 0.05) | 0.73 |
| NO_2_ | 1.12 (1.08-1.15) | 1.17 (1.13-1.20) | 1.04 (1.00-1.09) | 0.052 | 0.04 (0.00, 0.09) | 0.06 |
| NO_x_ | 1.12 (1.09-1.16) | 1.16 (1.13-1.20) | 1.04 (1.00-1.09) | 0.08 | 0.05 (0.00, 0.09) | 0.051 |
| **COPD** |  |  |  |  |  |  |
| PM_2.5_ absorbance | 1.21 (1.16-1.27) | 1.23 (1.18-1.28) | 1.01 (0.96-1.07) | 0.62 | 0.01 (-0.06, 0.07) | 0.83 |
| PM_2.5_ | 1.19 (1.14-1.25) | 1.24 (1.20-1.29) | 1.04 (0.99-1.11) | 0.14 | 0.06 (0.00, 0.13) | 0.053 |
| PM_coarse_ | 1.22 (1.17-1.27) | 1.22 (1.18-1.27) | 1.00 (0.94-1.06) | 0.99 | -0.01 (-0.07, 0.06) | 0.86 |
| PM_10_ | 1.21 (1.16-1.27) | 1.23 (1.18-1.28) | 1.02 (0.96-1.08) | 0.61 | 0.01 (-0.05, 0.07) | 0.80 |
| NO_2_ | 1.22 (1.17-1.27) | 1.23 (1.18-1.27) | 1.01 (0.95-1.07) | 0.81 | 0.01 (-0.05, 0.08) | 0.73 |
| **NO_x_** | **1.18 (1.13-1.23)** | **1.26 (1.21-1.31)** | **1.07 (1.01-1.13)** | **0.03** | **0.08 (0.02, 0.15)** | **0.009** |
| **Type 2 diabetes** |  |  |  |  |  |  |
| PM_2.5_ absorbance | 1.41 (1.37-1.46) | 1.38 (1.34-1.42) | 0.98 (0.93-1.02) | 0.30 | -0.04 (-0.10, 0.01) | 0.11 |
| PM_2.5_ | 1.41 (1.37-1.46) | 1.37 (1.33-1.42) | 0.97 (0.93-1.02) | 0.24 | 0.00 (-0.06, 0.05) | 0.87 |
| PM_coarse_ | 1.38 (1.33-1.42) | 1.41 (1.36-1.45) | 1.02 (0.98-1.07) | 0.35 | 0.02 (-0.04, 0.07) | 0.55 |
| PM_10_ | 1.39 (1.34-1.43) | 1.40 (1.36-1.44) | 1.01 (0.96-1.06) | 0.69 | 0.01 (-0.05, 0.06) | 0.85 |
| **NO_2_** | **1.44 (1.39-1.49)** | **1.36 (1.32-1.40)** | **0.95 (0.90-0.99)** | **0.02** | **-0.06 (-0.12, 0.00)** | **0.03** |
| NO_x_ | 1.40 (1.36-1.45) | 1.38 (1.34-1.42) | 0.98 (0.94-1.03) | 0.50 | 0.01 (-0.05, 0.06) | 0.78 |
| **All cancer** |  |  |  |  |  |  |
| **PM_2.5_ absorbance** | **1.03 (1.01-1.05)** | **1.07 (1.04-1.09)** | **1.04 (1.01-1.07)** | **0.01** | **0.04 (0.01, 0.07)** | **0.01** |
| **PM_2.5_** | **1.02 (1.00-1.05)** | **1.07 (1.05-1.09)** | **1.04 (1.01-1.07)** | **0.006** | **0.04 (0.01, 0.07)** | **0.005** |
| PM_coarse_ | 1.05 (1.02-1.07) | 1.04 (1.02-1.07) | 1.00 (0.97-1.03) | 0.91 | 0.00 (-0.03, 0.03) | 0.92 |
| PM_10_ | 1.04 (1.02-1.06) | 1.05 (1.03-1.07) | 1.01 (0.98-1.04) | 0.45 | 0.01 (-0.02, 0.04) | 0.44 |
| **NO_2_** | **1.02 (1.00-1.05)** | **1.07 (1.05-1.09)** | **1.04 (1.01-1.07)** | **0.006** | **0.04 (0.01, 0.07)** | **0.005** |
| **NO_x_** | **1.02 (1.00-1.04)** | **1.07 (1.05-1.10)** | **1.05 (1.02-1.09)** | **0.0004** | **0.05 (0.02, 0.08)** | **0.0004** |

Analyses adjusted for age, sex, ethnicity, deprivation index, smoking status, alcohol consumption, diet quality.

Table S8. Association between accelerometer-measured lower physical activity level and health outcomes by air pollution level in complete case analysis

|  | **HR stratified by air pollution level** | | **Interaction** | | | |
| --- | --- | --- | --- | --- | --- | --- |
|  | **Lower** | **Higher** | **HR_higher_ / HR_lower_** | **P** | **RERI** | **P** |
| **All-cause mortality** |  |  |  |  |  |  |
| PM_2.5_ absorbance | 1.53 (1.48-1.59) | 1.53 (1.48-1.59) | 1.00 (0.95-1.05) | 0.97 | -0.01 (-0.07, 0.05) | 0.71 |
| PM_2.5_ | 1.53 (1.48-1.59) | 1.53 (1.47-1.58) | 1.00 (0.95-1.05) | 0.86 | 0.02 (-0.04, 0.08) | 0.49 |
| PM_coarse_ | 1.53 (1.47-1.58) | 1.54 (1.48-1.60) | 1.01 (0.96-1.06) | 0.78 | -0.01 (-0.07, 0.05) | 0.66 |
| PM_10_ | 1.54 (1.49-1.60) | 1.53 (1.47-1.58) | 0.99 (0.94-1.04) | 0.77 | -0.03 (-0.09, 0.03) | 0.32 |
| NO_2_ | 1.53 (1.47-1.59) | 1.54 (1.48-1.59) | 1.00 (0.96-1.06) | 0.85 | 0.01 (-0.05, 0.07) | 0.79 |
| NO_x_ | 1.53 (1.48-1.59) | 1.53 (1.48-1.59) | 1.00 (0.95-1.05) | 0.98 | 0.02 (-0.04, 0.08) | 0.50 |
| **MACE** |  |  |  |  |  |  |
| PM_2.5_ absorbance | 1.35 (1.30-1.40) | 1.39 (1.34-1.45) | 1.03 (0.98-1.09) | 0.25 | 0.02 (-0.04, 0.07) | 0.60 |
| PM_2.5_ | 1.35 (1.30-1.40) | 1.39 (1.34-1.44) | 1.03 (0.97-1.08) | 0.35 | 0.05 (-0.01, 0.11) | 0.11 |
| PM_coarse_ | 1.35 (1.30-1.40) | 1.39 (1.34-1.44) | 1.03 (0.98-1.08) | 0.30 | 0.03 (-0.03, 0.08) | 0.40 |
| PM_10_ | 1.37 (1.32-1.42) | 1.38 (1.33-1.43) | 1.01 (0.95-1.06) | 0.83 | 0.00 (-0.06, 0.06) | 0.98 |
| NO_2_ | 1.35 (1.30-1.40) | 1.40 (1.35-1.45) | 1.04 (0.98-1.09) | 0.19 | 0.03 (-0.03, 0.09) | 0.27 |
| NO_x_ | 1.35 (1.30-1.40) | 1.39 (1.34-1.44) | 1.03 (0.98-1.09) | 0.24 | 0.04 (-0.02, 0.10) | 0.15 |
| **COPD** |  |  |  |  |  |  |
| PM_2.5_ absorbance | 1.35 (1.28-1.42) | 1.29 (1.23-1.36) | 0.96 (0.89-1.03) | 0.23 | -0.06 (-0.14, 0.02) | 0.15 |
| PM_2.5_ | 1.34 (1.27-1.42) | 1.29 (1.23-1.36) | 0.96 (0.90-1.03) | 0.30 | -0.02 (-0.11, 0.06) | 0.57 |
| PM_coarse_ | 1.33 (1.26-1.40) | 1.31 (1.25-1.38) | 0.99 (0.92-1.06) | 0.80 | -0.02 (-0.10, 0.06) | 0.60 |
| PM_10_ | 1.34 (1.27-1.41) | 1.30 (1.24-1.37) | 0.97 (0.90-1.04) | 0.42 | -0.05 (-0.13, 0.03) | 0.26 |
| NO_2_ | 1.35 (1.28-1.42) | 1.30 (1.23-1.36) | 0.96 (0.89-1.03) | 0.29 | -0.04 (-0.13, 0.04) | 0.32 |
| NO_x_ | 1.35 (1.28-1.43) | 1.29 (1.23-1.36) | 0.96 (0.89-1.03) | 0.23 | -0.04 (-0.12, 0.05) | 0.41 |
| **Type 2 diabetes** |  |  |  |  |  |  |
| PM_2.5_ absorbance | 1.78 (1.71-1.86) | 1.73 (1.66-1.81) | 0.97 (0.91-1.03) | 0.34 | -0.09 (-0.17, 0.01) | 0.03 |
| PM_2.5_ | 1.78 (1.70-1.87) | 1.72 (1.65-1.80) | 0.97 (0.91-1.03) | 0.28 | -0.01 (-0.09, 0.07) | 0.82 |
| PM_coarse_ | 1.76 (1.68-1.84) | 1.75 (1.67-1.82) | 0.99 (0.93-1.05) | 0.80 | -0.03 (-0.11, 0.05) | 0.48 |
| PM_10_ | 1.79 (1.71-1.87) | 1.72 (1.65-1.80) | 0.97 (0.91-1.03) | 0.26 | -0.06 (-0.14, 0.02) | 0.15 |
| NO_2_ | 1.80 (1.72-1.88) | 1.72 (1.65-1.79) | 0.95 (0.90-1.01) | 0.13 | -0.07 (-0.15, 0.02) | 0.11 |
| NO_x_ | 1.78 (1.70-1.86) | 1.73 (1.66-1.81) | 0.97 (0.92-1.04) | 0.41 | 0.00 (-0.09, 0.08) | 0.96 |
| **All cancer** |  |  |  |  |  |  |
| PM_2.5_ absorbance | 1.13 (1.10-1.15) | 1.14 (1.11-1.17) | 1.02 (0.98-1.05) | 0.43 | 0.02 (-0.02, 0.06) | 0.41 |
| PM_2.5_ | 1.12 (1.09-1.15) | 1.15 (1.11-1.18) | 1.02 (0.98-1.06) | 0.27 | 0.02 (-0.02, 0.06) | 0.23 |
| PM_coarse_ | 1.14 (1.11-1.17) | 1.13 (1.10-1.16) | 0.99 (0.96-1.03) | 0.77 | -0.01 (-0.05, 0.03) | 0.76 |
| PM_10_ | 1.13 (1.10-1.16) | 1.14 (1.11-1.17) | 1.01 (0.97-1.04) | 0.79 | 0.01 (-0.03, 0.04) | 0.79 |
| NO_2_ | 1.12 (1.09-1.15) | 1.15 (1.12-1.18) | 1.02 (0.98-1.06) | 0.28 | 0.02 (-0.02, 0.06) | 0.30 |
| NO_x_ | 1.13 (1.10-1.15) | 1.14 (1.11-1.17) | 1.02 (0.98-1.05) | 0.42 | 0.02 (-0.02, 0.05) | 0.43 |

Analyses adjusted for age, sex, ethnicity, deprivation index, smoking status, alcohol consumption, diet quality.

Table S9. Association between per IQR decrease in self-reported physical activity level and health outcomes by air pollution level

|  | **HR of PA_SR_ by air pollution level** | | **Interaction** | | | |
| --- | --- | --- | --- | --- | --- | --- |
|  | **1 IQR lower** | **1 IQR higher** | **HR_higher_ / HR_lower_** | **P** | **RERI** | **P** |
| **All-cause mortality** |  |  |  |  |  |  |
| PM_2.5_ absorbance | 1.09 (0.97-1.23) | 1.09 (0.92-1.28) | 1.00 (0.95-1.04) | 0.90 | -0.01 (-0.05, 0.03) | 0.61 |
| PM_2.5_ | 1.09 (1.06-1.12) | 1.09 (1.07-1.12) | 1.00 (0.99-1.01) | 0.75 | 0.00 (-0.01, 0.02) | 0.53 |
| PM_course_ | 1.09 (1.07-1.12) | 1.09 (1.06-1.13) | 1.00 (0.98-1.01) | 0.74 | 0.00 (-0.02, 0.01) | 0.65 |
| PM_10_ | 1.10 (1.05-1.15) | 1.10 (1.05-1.15) | 1.00 (0.99-1.01) | 0.93 | 0.00 (-0.01, 0.01) | 0.93 |
| NO_2_ | 1.07 (1.03-1.12) | 1.08 (1.03-1.12) | 1.00 (1.00-1.00) | 0.29 | 0.00 (0.00, 0.00) | 0.25 |
| NO_x_ | 1.08 (1.04-1.12) | 1.08 (1.04-1.12) | 1.00 (1.00-1.00) | 0.34 | 0.00 (0.00, 0.00) | 0.22 |
| **MACE** |  |  |  |  |  |  |
| PM_2.5_ absorbance | 1.07 (0.95-1.20) | 1.07 (0.91-1.26) | 1.00 (0.96-1.05) | 0.84 | 0.00 (-0.03, 0.02) | 0.78 |
| PM_2.5_ | 1.03 (1.00-1.06) | 1.04 (1.02-1.06) | 1.01 (1.00-1.02) | 0.08 | 0.01 (0.00, 0.02) | 0.052 |
| PM_course_ | 1.06 (1.04-1.08) | 1.06 (1.03-1.09) | 1.00 (0.99-1.02) | 0.69 | 0.00 (-0.01, 0.02) | 0.67 |
| PM_10_ | 1.04 (1.00-1.09) | 1.04 (1.00-1.09) | 1.00 (1.00-1.01) | 0.64 | 0.00 (0.00, 0.01) | 0.59 |
| NO_2_ | 1.02 (0.98-1.06) | 1.02 (0.98-1.06) | 1.00 (1.00-1.00) | 0.08 | 0.00 (0.00, 0.00) | 0.09 |
| NO_x_ | 1.03 (1.00-1.07) | 1.03 (1.00-1.07) | 1.00 (1.00-1.00) | 0.22 | 0.00 (0.00, 0.00) | 0.17 |
| **COPD** |  |  |  |  |  |  |
| PM_2.5_ absorbance | 1.16 (1.00-1.35) | 1.20 (0.98-1.48) | 1.03 (0.98-1.09) | 0.26 | 0.01 (-0.03, 0.05) | 0.71 |
| PM_2.5_ | 1.05 (1.01-1.09) | 1.06 (1.03-1.08) | 1.01 (0.99-1.02) | 0.34 | 0.01 (0.00, 0.02) | 0.19 |
| PM_course_ | 1.09 (1.06-1.12) | 1.10 (1.06-1.15) | 1.01 (1.00-1.03) | 0.10 | 0.02 (0.00, 0.04) | 0.13 |
| PM_10_ | 1.00 (0.94-1.07) | 1.01 (0.96-1.07) | 1.01 (1.00-1.02) | 0.04 | 0.01 (0.00, 0.02) | 0.02 |
| NO_2_ | 1.04 (0.99-1.09) | 1.04 (0.99-1.09) | 1.00 (1.00-1.00) | 0.27 | 0.00 (0.00, 0.00) | 0.26 |
| NO_x_ | 1.03 (0.99-1.08) | 1.04 (0.99-1.08) | 1.00 (1.00-1.00) | 0.13 | 0.00 (0.00, 0.00) | 0.08 |
| **Type 2 diabetes** |  |  |  |  |  |  |
| **PM_2.5_ absorbance** | **1.09 (0.97-1.23)** | **1.06 (0.89-1.26)** | **0.97 (0.92-1.02)** | **0.21** | **-0.04 (-0.07, -0.01)** | **0.004** |
| PM_2.5_ | 1.19 (1.15-1.23) | 1.19 (1.16-1.21) | 1.00 (0.98-1.01) | 0.54 | 0.00 (-0.01, 0.02) | 0.89 |
| PM_course_ | 1.20 (1.17-1.22) | 1.21 (1.17-1.25) | 1.01 (1.00-1.03) | 0.13 | 0.02 (0.00, 0.03) | 0.12 |
| PM_10_ | 1.14 (1.08-1.20) | 1.14 (1.09-1.20) | 1.01 (1.00-1.01) | 0.14 | 0.01 (0.00, 0.01) | 0.055 |
| NO_2_ | 1.22 (1.17-1.27) | 1.22 (1.17-1.27) | 1.00 (1.00-1.00) | 0.13 | 0.00 (0.00, 0.00) | 0.12 |
| NO_x_ | 1.20 (1.16-1.25) | 1.20 (1.16-1.24) | 1.00 (1.00-1.00) | 0.29 | 0.00 (0.00, 0.00) | 0.59 |
| **All cancer** |  |  |  |  |  |  |
| PM_2.5_ absorbance | 1.03 (0.95-1.11) | 1.03 (0.92-1.15) | 1.00 (0.97-1.03) | 1.00 | 0.00 (-0.03, 0.03) | 0.99 |
| PM_2.5_ | 1.01 (1.00-1.03) | 1.02 (1.01-1.03) | 1.00 (1.00-1.01) | 0.20 | 0.01 (0.00, 0.01) | 0.18 |
| PM_course_ | 1.03 (1.01-1.04) | 1.03 (1.00-1.05) | 1.00 (0.99-1.01) | 0.97 | 0.00 (-0.01, 0.01) | 0.98 |
| PM_10_ | 1.02 (0.99-1.05) | 1.02 (0.99-1.05) | 1.00 (1.00-1.00) | 0.74 | 0.00 (0.00, 0.01) | 0.71 |
| NO_2_ | 1.01 (0.99-1.04) | 1.01 (0.99-1.04) | 1.00 (1.00-1.00) | 0.40 | 0.00 (0.00, 0.00) | 0.38 |
| NO_x_ | 1.01 (0.99-1.04) | 1.01 (0.99-1.04) | 1.00 (1.00-1.00) | 0.31 | 0.00 (0.00, 0.00) | 0.29 |

Analyses adjusted for age, sex, ethnicity, deprivation index, smoking status, alcohol consumption, diet quality.

Table S10. Association between per IQR decrease in accelerometer-measured physical activity level and health outcomes by air pollution level

|  | **HR of PA_ACC_ by air pollution level** | | **Interaction** | | | |
| --- | --- | --- | --- | --- | --- | --- |
|  | **1 IQR lower** | **1 IQR higher** | **HR_higher_ / HR_lower_** | **P** | **RERI** | **P** |
| **All-cause mortality** |  |  |  |  |  |  |
| **PM_2.5_ absorbance** | **1.41 (1.20-1.65)** | **1.40 (1.12-1.75)** | **1.00 (0.94-1.06)** | **0.95** | **-0.06 (-0.10, -0.02)** | **0.007** |
| PM_2.5_ | 1.43 (1.37-1.48) | 1.42 (1.38-1.46) | 0.99 (0.98-1.01) | 0.45 | 0.00 (-0.02, 0.02) | 0.81 |
| PM_course_ | 1.42 (1.38-1.46) | 1.43 (1.36-1.49) | 1.00 (0.99-1.02) | 0.62 | 0.00 (-0.03-0.02) | 1.00 |
| PM_10_ | 1.42 (1.33-1.51) | 1.41 (1.34-1.50) | 1.00 (0.99-1.01) | 0.92 | 0.00 (-0.01, 0.01) | 0.72 |
| NO_2_ | 1.42 (1.35-1.50) | 1.42 (1.35-1.50) | 1.00 (1.00-1.00) | 0.73 | 0.00 (0.00, 0.00) | 0.53 |
| NO_x_ | 1.43 (1.36-1.50) | 1.43 (1.36-1.49) | 1.00 (1.00-1.00) | 0.55 | 0.00 (0.00, 0.00) | 0.90 |
| **MACE** |  |  |  |  |  |  |
| PM_2.5_ absorbance | 1.40 (1.18-1.65) | 1.45 (1.15-1.82) | 1.03 (0.97-1.10) | 0.31 | -0.02 (-0.07, 0.02) | 0.32 |
| PM_2.5_ | 1.26 (1.21-1.31) | 1.27 (1.24-1.30) | 1.01 (0.99-1.02) | 0.35 | 0.01 (0.00, 0.03) | 0.14 |
| PM_course_ | 1.29 (1.25-1.33) | 1.30 (1.24-1.36) | 1.00 (0.99-1.02) | 0.63 | 0.01 (-0.02, 0.03) | 0.58 |
| PM_10_ | 1.25 (1.17-1.34) | 1.26 (1.19-1.33) | 1.00 (0.99-1.01) | 0.50 | 0.00 (-0.01, 0.01) | 0.46 |
| NO_2_ | 1.25 (1.18-1.32) | 1.25 (1.18-1.32) | 1.00 (1.00-1.00) | 0.28 | 0.00 (0.00, 0.00) | 0.58 |
| NO_x_ | 1.26 (1.20-1.32) | 1.26 (1.20-1.32) | 1.00 (1.00-1.00) | 0.35 | 0.00 (0.00, 0.00) | 0.25 |
| **COPD** |  |  |  |  |  |  |
| PM_2.5_ absorbance | 1.27 (1.02-1.57) | 1.28 (0.95-1.72) | 1.01 (0.93-1.10) | 0.83 | -0.03 (-0.08, 0.02) | 0.23 |
| PM_2.5_ | 1.26 (1.19-1.33) | 1.25 (1.20-1.30) | 0.99 (0.97-1.01) | 0.38 | 0.00 (-0.03, 0.02) | 0.90 |
| PM_course_ | 1.25 (1.20-1.30) | 1.26 (1.18-1.34) | 1.01 (0.98-1.04) | 0.45 | 0.01 (-0.02, 0.04) | 0.63 |
| PM_10_ | 1.23 (1.12-1.35) | 1.23 (1.13-1.33) | 1.00 (0.99-1.01) | 0.95 | 0.00 (-0.01, 0.02) | 0.95 |
| NO_2_ | 1.26 (1.17-1.36) | 1.26 (1.17-1.35) | 1.00 (1.00-1.00) | 0.6 | 0.00 (0.00, 0.00) | 0.57 |
| NO_x_ | 1.25 (1.17-1.33) | 1.25 (1.17-1.33) | 1.00 (1.00-1.00) | 0.63 | 0.00 (0.00, 0.00) | 0.99 |
| **Type 2 diabetes** |  |  |  |  |  |  |
| **PM_2.5_ absorbance** | **1.38 (1.14-1.66)** | **1.32 (1.02-1.70)** | **0.95 (0.89-1.03)** | **0.21** | **-0.11 (-0.14, -0.07)** | **< 0.0001** |
| PM_2.5_ | 1.59 (1.51-1.67) | 1.57 (1.52-1.62) | 0.99 (0.97-1.01) | 0.27 | -0.01 (-0.04, 0.02) | 0.61 |
| PM_course_ | 1.56 (1.50-1.61) | 1.56 (1.48-1.64) | 1.00 (0.98-1.03) | 0.85 | 0.00 (-0.03, 0.04) | 0.78 |
| PM_10_ | 1.61 (1.49-1.75) | 1.60 (1.49-1.72) | 0.99 (0.98-1.01) | 0.33 | -0.01 (-0.02, 0.01) | 0.52 |
| NO_2_ | 1.64 (1.53-1.75) | 1.63 (1.53-1.74) | 1.00 (1.00-1.00) | 0.1 | -0.01 (-0.01, 0.00) | 0.02 |
| NO_x_ | 1.60 (1.51-1.69) | 1.60 (1.51-1.69) | 1.00 (1.00-1.00) | 0.27 | 0.00 (0.00, 0.00) | 0.42 |
| **All cancer** |  |  |  |  |  |  |
| PM_2.5_ absorbance | 1.18 (1.05-1.32) | 1.21 (1.03-1.42) | 1.03 (0.98-1.08) | 0.21 | 0.03 (-0.02, 0.09) | 0.27 |
| PM_2.5_ | 1.08 (1.05-1.10) | 1.08 (1.06-1.10) | 1.01 (1.00-1.02) | 0.15 | 0.01 (0.00, 0.02) | 0.11 |
| PM_course_ | 1.10 (1.07-1.12) | 1.10 (1.06-1.13) | 1.00 (0.99-1.01) | 0.87 | 0.00 (-0.01, 0.02) | 0.82 |
| PM_10_ | 1.08 (1.03-1.13) | 1.08 (1.04-1.13) | 1.00 (1.00-1.01) | 0.65 | 0.00 (0.00, 0.01) | 0.57 |
| NO_2_ | 1.06 (1.02-1.10) | 1.06 (1.02-1.10) | 1.00 (1.00-1.00) | 0.07 | 0.00 (0.00, 0.00) | 0.055 |
| NO_x_ | 1.07 (1.03-1.11) | 1.07 (1.04-1.11) | 1.00 (1.00-1.00) | 0.15 | 0.00 (0.00, 0.00) | 0.12 |

Analyses adjusted for age, sex, ethnicity, deprivation index, smoking status, alcohol consumption, diet quality.

Table S11. Association between lower self-reported physical activity level and health outcomes by air pollution level among people with valid accelerometer data

|  | **HR stratified by air pollution level** | | **Interaction** | | | |
| --- | --- | --- | --- | --- | --- | --- |
|  | **Lower** | **Higher** | **HR_higher_ / HR_lower_** | **P** | **RERI** | **P** |
| **All-cause mortality** |  |  |  |  |  |  |
| PM_2.5_ absorbance | 1.15 (1.04-1.26) | 1.20 (1.09-1.33) | 1.05 (0.92-1.20) | 0.48 | 0.06 (-0.09-0.20) | 0.45 |
| PM_2.5_ | 1.17 (1.07-1.28) | 1.17 (1.06-1.30) | 1.00 (0.88-1.15) | 0.95 | 0.01 (-0.14-0.16) | 0.92 |
| PM_coarse_ | 1.21 (1.10-1.33) | 1.13 (1.02-1.25) | 0.93 (0.81-1.07) | 0.30 | -0.08 (-0.23-0.07) | 0.30 |
| PM_10_ | 1.20 (1.09-1.32) | 1.15 (1.04-1.26) | 0.96 (0.84-1.10) | 0.55 | -0.04 (-0.20-0.11) | 0.56 |
| NO_2_ | 1.13 (1.03-1.24) | 1.22 (1.11-1.35) | 1.08 (0.94-1.24) | 0.27 | 0.08 (-0.06-0.23) | 0.25 |
| NO_x_ | 1.12 (1.02-1.23) | 1.23 (1.11-1.35) | 1.09 (0.95-1.25) | 0.20 | 0.11 (-0.04-0.25) | 0.15 |
| **MACE** |  |  |  |  |  |  |
| PM_2.5_ absorbance | 1.07 (0.99-1.16) | 1.07 (0.98-1.16) | 1.00 (0.89-1.12) | 0.95 | -0.01 (-0.12-0.11) | 0.93 |
| PM_2.5_ | 1.04 (0.96-1.12) | 1.11 (1.02-1.20) | 1.07 (0.95-1.19) | 0.26 | 0.07 (-0.05-0.19) | 0.23 |
| PM_coarse_ | 1.03 (0.95-1.11) | 1.12 (1.03-1.21) | 1.09 (0.97-1.21) | 0.16 | 0.08 (-0.03-0.19) | 0.15 |
| **PM_10_** | **1.00 (0.93-1.09)** | **1.15 (1.06-1.24)** | **1.14 (1.02-1.28)** | **0.02** | **0.13 (0.02-0.24)** | **0.02** |
| NO_2_ | 1.03 (0.95-1.11) | 1.13 (1.04-1.22) | 1.10 (0.98-1.23) | 0.11 | 0.09 (-0.02-0.20) | 0.11 |
| **NO_x_** | **1.02 (0.94-1.10)** | **1.14 (1.05-1.24)** | **1.12 (1.00-1.25)** | **0.05** | **0.12 (0.00-0.23)** | **0.04** |
| **COPD** |  |  |  |  |  |  |
| PM_2.5_ absorbance | 1.30 (1.16-1.45) | 1.27 (1.13-1.42) | 0.98 (0.83-1.15) | 0.77 | -0.04 (-0.22-0.14) | 0.63 |
| PM_2.5_ | 1.26 (1.12-1.41) | 1.31 (1.17-1.46) | 1.04 (0.89-1.22) | 0.64 | 0.05 (-0.13-0.23) | 0.59 |
| PM_coarse_ | 1.27 (1.14-1.43) | 1.29 (1.15-1.44) | 1.01 (0.86-1.19) | 0.89 | 0.00 (-0.18-0.18) | 0.99 |
| PM_10_ | 1.20 (1.07-1.35) | 1.36 (1.22-1.53) | 1.14 (0.97-1.33) | 0.12 | 0.12 (-0.04-0.29) | 0.14 |
| NO_2_ | 1.30 (1.16-1.46) | 1.26 (1.13-1.41) | 0.97 (0.83-1.14) | 0.73 | -0.04 (-0.22-0.15) | 0.69 |
| NO_x_ | 1.25 (1.11-1.40) | 1.31 (1.18-1.47) | 1.05 (0.90-1.24) | 0.51 | 0.07 (-0.11-0.24) | 0.47 |
| **Type 2 diabetes** |  |  |  |  |  |  |
| PM_2.5_ absorbance | 1.39 (1.28-1.52) | 1.31 (1.20-1.42) | 0.94 (0.83-1.06) | 0.29 | -0.09 (-0.23-0.05) | 0.19 |
| PM_2.5_ | 1.34 (1.24-1.46) | 1.36 (1.25-1.47) | 1.01 (0.90-1.13) | 0.90 | 0.04 (-0.11-0.18) | 0.61 |
| PM_coarse_ | 1.34 (1.23-1.46) | 1.36 (1.26-1.48) | 1.02 (0.91-1.15) | 0.74 | 0.02 (-0.12-0.15) | 0.80 |
| PM_10_ | 1.39 (1.27-1.51) | 1.32 (1.22-1.43) | 0.95 (0.85-1.07) | 0.43 | -0.06 (-0.20-0.08) | 0.42 |
| NO_2_ | 1.39 (1.28-1.52) | 1.31 (1.21-1.43) | 0.94 (0.84-1.06) | 0.33 | -0.06 (-0.21-0.08) | 0.40 |
| NO_x_ | 1.36 (1.25-1.48) | 1.34 (1.24-1.46) | 0.99 (0.88-1.12) | 0.88 | 0.02 (-0.12-0.16) | 0.80 |
| **All cancer** |  |  |  |  |  |  |
| PM_2.5_ absorbance | 1.02 (0.98-1.07) | 1.06 (1.01-1.11) | 1.03 (0.97-1.11) | 0.35 | 0.03 (-0.04-0.10) | 0.34 |
| PM_2.5_ | 1.03 (0.98-1.08) | 1.05 (1.00-1.11) | 1.03 (0.96-1.10) | 0.44 | 0.03 (-0.04-0.10) | 0.44 |
| PM_coarse_ | 1.06 (1.01-1.11) | 1.02 (0.97-1.07) | 0.96 (0.90-1.03) | 0.26 | -0.04 (-0.11-0.03) | 0.28 |
| PM_10_ | 1.06 (1.01-1.11) | 1.02 (0.97-1.07) | 0.96 (0.90-1.03) | 0.25 | -0.04 (-0.11-0.03) | 0.26 |
| NO_2_ | 1.02 (0.98-1.07) | 1.06 (1.01-1.11) | 1.03 (0.96-1.10) | 0.37 | 0.03 (-0.04-0.10) | 0.37 |
| NO_x_ | 1.01 (0.97-1.06) | 1.07 (1.02-1.13) | 1.06 (0.99-1.13) | 0.09 | 0.06 (-0.01-0.13) | 0.09 |

Analyses adjusted for age, sex, ethnicity, deprivation index, smoking status, alcohol consumption, diet quality.
